# Implementation of a community-based low-calorie dietary intervention for the induction of type-2 diabetes and pre-diabetes remission: a feasibility study utilising a type 2 hybrid design

**DOI:** 10.1186/s43058-021-00196-9

**Published:** 2021-08-28

**Authors:** Kim R. Quimby, Natasha Sobers, Colette George, Natalie Greaves, Francine Browman-Jones, T. Alafia Samuels

**Affiliations:** 1 George Alleyne Chronic Disease Research Centre, Caribbean Institute for Health Research, The University of the West Indies, Jemmott’s Lane, St. Michael, Bridgetown, Barbados; 2 Faculty of Medical Sciences, The University of the West Indies, Cave Hill Campus, Bridgetown, Barbados; 3 Ministry of Health of Barbados, Bridgetown, Barbados; 4 Epidemiology Research Unit, Caribbean Institute for Health research, The University of the West Indies, Kingston, Jamaica

**Keywords:** Type 2 diabetes, Diabetes remission, Weight loss, Community intervention, Community health advocates, Faith-based organisation, Low-calorie diet, Overweight

## Abstract

**Objectives:**

The aims of this feasibility study were to (1) examine the implementation of a community-based health advocate (CHA) training programme to develop the clinical skills needed to support a diabetes remission protocol based on a low-calorie diet (LCD) and (2) investigate if participant weight loss can be achieved and diabetes remission induced under these conditions.

**Methods:**

This tripartite study followed a type 2 implementation-effectiveness design. Three faith-based organisations (FBOs) were purposively selected as study sites. Implementation outcomes were guided by the Consolidated Framework for Implementation Research. During the pre-implementation phase, site ‘readiness’ to facilitate the intervention was determined from a site visit and an interview with the FBOs’ leadership. During the implementation phase, congregants could volunteer for the 10-week CHA training which included practical exercises in weight, glucose and blood pressure (BP) measurement, and a summative practical assessment. Acceptability and implementation effectiveness were assessed via survey. During the intervention phase, other congregants and community members with T2DM or pre-diabetes and overweight were invited to participate in the 12-week LCD. Anti-diabetic medication was discontinued on day 1 of the intervention. Clinical effectiveness was determined from the change in weight, fasting blood glucose (FBG) and BP which were monitored weekly at the FBO by the CHA. HbA1C was performed at weeks 1 and 12.

**Results:**

The FBOs were found to be ready as determined by their adequate resources and engagement in health-related matters. Twenty-nine CHAs completed the training; all attained a passing grade at ≥1 clinical station, indicating implementation effectiveness. CHA feedback indicated that the programme structure was acceptable and provided sufficient access to intervention-related material. Thirty-one persons participated in the LCD (11 T2DM:20 pre-diabetes). Mean (95%CI) weight loss was 6.0 kg (3.7 to 8.2), 7.9 kg in males vs 5.7 kg in females; A1C (%) decreased from 6.6 to 6.1, with a greater reduction in those with T2DM when compared to pre-diabetes. FBG decreased from 6.4 to 6.0mmol/L. T2DM remission rates were 60% and 90% by A1C<6.5% and FBG<7mmol/L respectively. Pre-diabetes remission was 18% and 40% by A1C<5.7% and FBG<5.6 respectively.

**Conclusion:**

Implementation of a community-based diabetes remission protocol is both feasible and clinically effective. Its sustainability is to be determined. Adaptability to other disorders or other settings should be investigated.

**Trial registration:**

NCT03536377 registered on 24 May 2018.

Contributions to the literature

- Weight loss-induced T2DM remission due to a low-calorie diet is feasible in clinical settings; however, its feasibility in a community-based approach is not yet established.
- This study shows that faith-based organisations are equipped to successfully support a community health advocates training programme aimed at increasing the skills necessary for T2DM screening and the effective delivery of a weight loss and T2DM remission protocol; moreover, weight loss and T2DM and pre-diabetes remission can be achieved under these conditions.
- We recommend community-based screening for T2DM and pre-diabetes and propose investigating the adaptability of this intervention to other community sites and disease states.

## Background

The 2019 International Diabetes Federation report estimated that 463 million adults are living with diabetes [1]. As with other non-communicable diseases (NCDs), the prevalence is highest in high-income countries, with North America and the Caribbean (NAC) bearing the highest burden. The prevalence of pre-diabetes is also increasing, and the 5-year conversion rate to type 2 diabetes mellitus (T2DM) ranges from 26 to 50%, influenced by a complex interplay of factors such as weight, age, race and an obesogenic environment that compromises the lifestyle measures necessary for T2DM control [2–7]. Likewise, obesity prevalence has nearly tripled in the last 50 years [8]. Within NAC, the prevalence of obesity was 28%, increasing to 62.5% when overweight data were included [9, 10].

Historically, T2DM has been viewed as a life-long illness; however, recent interventions are shifting this perception. Weight loss as a result of bariatric surgery has led to the concept of ‘diabetes remission’ as defined by the restoration of β cell function and insulin sensitivity and the resultant normalisation of blood glucose; benefits that have been reportedly maintained for up to 10 years post-operatively [11, 12]. The widespread impact of bariatric surgery, however, is limited by its eligibility criteria which in the Swedish Bariatric cohort was restricted to men with at least class I and women with at least class II obesity [12]. The low-calorie dietary intervention (LCD) which is defined by a daily caloric intake of ≤ 1200 Kcal offers an alternative approach which increases the eligibility profile while producing similar improvements in metabolic outcome. The Counterpoint LCD trial employed a case-control study design where participants were monitored on a weekly basis at the clinic-based study site. At the end of the 8-week period, those on the calorie-restricted diet achieved significant improvements in glucose control as compared to the control arm [13]. We have reproduced this trial in our local, clinic-based Barbados Diabetes Reversal Study-1 (BDRS1), to confirm that the metabolic effects achieved in a predominantly Black population are comparable with those in the predominantly White population where the protocol was originally published [14]. The DiReCT trial expanded the intervention locations to include primary care practices. Participants remained on the 600Kcal diet for 12–20 weeks. At the 1-year follow-up, the percentage of participants who maintained diabetes remission was influenced by the percentage weight loss during the intervention period. This ranged from 7% still in remission amongst those who lost up to 5kg to 86% remission in those who lost ≥15kgs. The success of this trial confirmed the transferability of this intervention to a routine clinical practice [15]. Although the treatment options for overweight and T2DM are increasing, disparities in access still exist [1]. Empowering community health advocates (CHA), that is, non-medical members of a community, is a useful implementation strategy in the advancement of community-based clinical interventions such as those promoting health education, physical activity and other lifestyle changes [16, 17]. In addition, this strategy has been shown to increase accessibility while effectively achieving health outcomes comparable to physician-led efforts including improved medication adherence, blood pressure readings and decreased depression scores [18, 19]. The use of faith-based organisations (FBOs) as community sites is particularly common in ethnic minority and underserved people groups; however, the implementation of a community-based LCD intervention for diabetes remission has not been previously explored [20, 21].

### The setting

Barbados is a 166-square mile Caribbean island with a population of approximately 280,000. Consistent with the epidemiologic profile of most small island developing states, non-communicable diseases (NCDs) are responsible for >80% of deaths and the risk of premature deaths from NCDs is approximately 16% [22]. While coronary heart disease is the number one cause of death, T2DM is the primary metabolic contributor to NCD deaths on the island.

The Barbados Health of the Nation 2012 study reported that 18.7% of the population had a diagnosis of T2DM; of these, 13.8% were unaware of their status and 66.7% were uncontrolled [23]. An additional 14.7% of the population had impaired fasting glucose and 66% were overweight inclusive of 33% who were obese. Understandably, diabetes-related complications consume a significant portion of healthcare expenditure [24]. The Barbados government, in adopting a whole-of-society approach to NCDs, has engaged faith-based organisations (FBOs), who now have representation on the island’s NCD commission [25]. This level of civil society engagement is in alignment with the Caribbean Community (CARICOM) strategy for combatting NCDs as articulated in the Port-of-Spain declaration [26]. Importantly, Barbados has a high density of FBOs, many of which are integrated into the surrounding communities, including functioning as community disaster shelters. Given this local context, FBOs may be viewed as being acceptable and accessible locations for the implementation of community-based interventions.

### Aim

The overarching aim of the Barbados Diabetes Remission Study 2 (BDRS2) was to investigate the feasibility of implementing a community-based low calorie dietary intervention (LCD) for the induction of T2DM and pre-diabetes remission. The approach included two distinct objectives (1) to assess the implementation of a community-based health advocate (CHA) training programme to develop the clinical skills needed to support a diabetes remission protocol based on a LCD and (2) to investigate if participant weight loss can be achieved and diabetes remission induced under these conditions. The objectives were framed using a type 2 implementation-effectiveness hybrid design which focusses equally on both implementation outcome, e.g. acceptability, and intervention outcomes, e.g. change in clinical measures [27].

## Methods

**Fig. 1:**
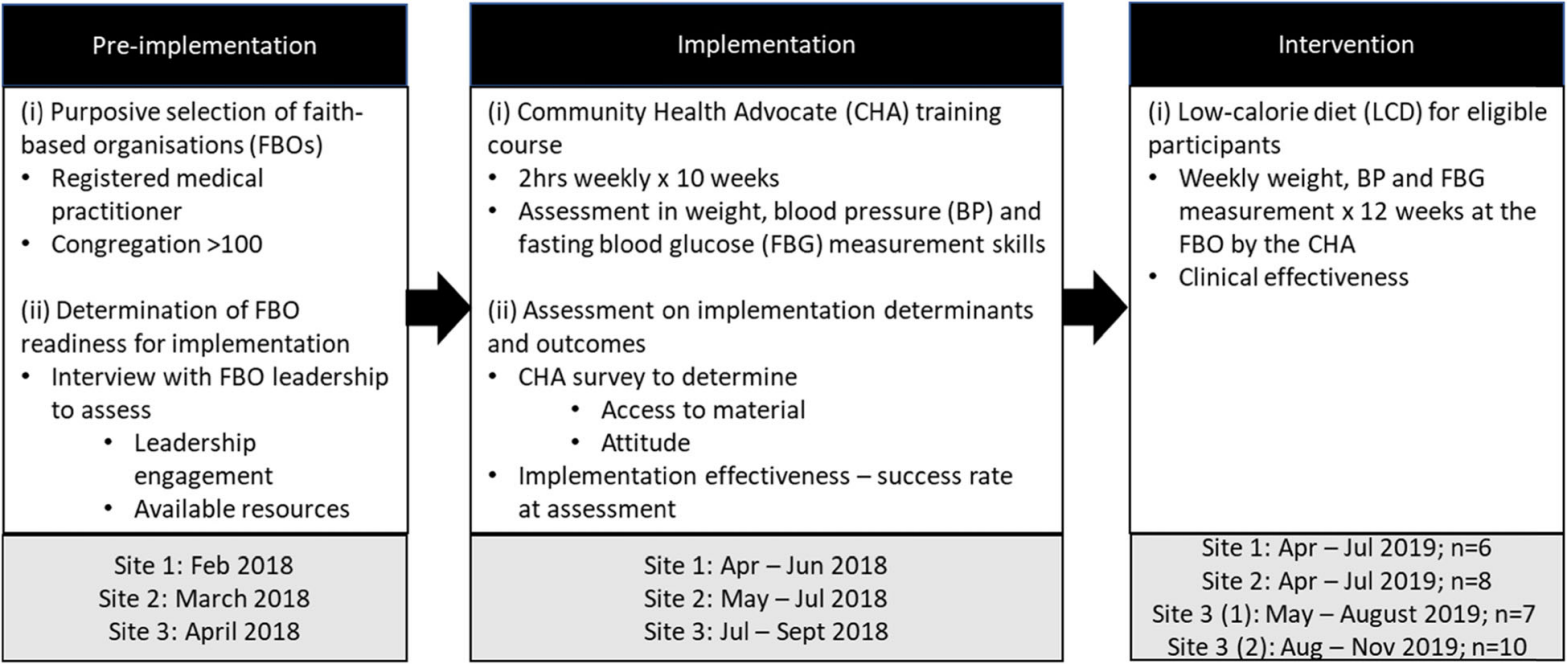
Pre-implementation, implementation and intervention phases of the BDRS2 study protocol

The protocol was carried out in three distinct steps: (1) the pre-implementation phase where the faith-based organisations (FBOs) were engaged, (2) the implementation phase which utilised an implementation strategy focused on increasing the knowledge and skills of community health advocates (CHA) to support the low-calorie diet (LCD) and (3) the intervention phase where eligible persons with T2DM or pre-diabetes and overweight participated in the LCD and were supported by the trained CHAs (Fig. 1) [28].

1. Pre-implementation

The pre-implementation process is presented in two steps: (i) purposive selection of FBOs and (ii) determination of readiness for implementation (Fig. 1).

(i) Purposive selection of the FBO: Three FBOs were purposively samples based on two main criteria: (a) the presence of a medical doctor as a part of the FBO membership who would be available to consult with the local study team and (b) an attendance of >100 persons at the FBO’s main worship service as this could provide a large enough sampling frame.
(ii) Determination of readiness for implementation: Readiness was defined as by the Consolidated Framework for Implementation Research (CFIR) [29]. It is an inner setting construct which encompasses three core elements: (1) leadership engagement, (2) the availability of resources and (3) access to intervention-related information.

Readiness was determined via an interview with the leadership of the three FBO sites during the months of February to April 2018. This interview was guided by a questionnaire that was adapted from the CONTACT study in Guyana [30]. It included 6 questions which determined leadership engagement—these measured the FBO’s leaders’ involvement in health- and intervention-related activities (Table 1). Another 6 questions assessed the available resources including both the physical space dedicated for implementation activities and the human resources—namely congregants who were willing to volunteer and to be trained as CHAs to deliver the intervention. In addition to the leader’s report of the availability of physical resources and health personnel, site visits were made by the Principal Investigator of the study to all FBOs to identify and verify the availability of spaces, and the study was introduced to the general congregation at major services and any adult member of the FBO was eligible to volunteer for the CHA training course; no previous training or experience was required.

**Table. 1:** The FBOs leaders’ estimation of the readiness of the FBOs to implement the intervention

Domains of readiness	Assessment question
Leadership engagement	1. Do you have teaching sessions on health-related issues? 2. Which health-related issues have been taught in the last year? 3. Does your place of worship have an ongoing programme for persons living with diabetes? 4. Does your place of worship have an ongoing programme to reduce risk factors for diabetes? E.g. health eating, active lifestyle (*Emphasis here on* *prevention*) 5. Does your congregation have any plans for implementing new, or improving on existing health-related activities? 6. Do you see a need for a diabetes health promotion and prevention programme within your congregation?
Available resources (physical space)	1. Do you have a small, enclosed room where the CHA can see members in private? 2. Do you have resources and facilities for secure and organised record keeping? 3. Do you have resources and facilities for cooking classes?
Available resources (human resources)	1. Does your congregation have someone who oversees health-related matters? 2. Does he/she have any formal qualifications for the position? E.g. health professional / work experience in the medical field 3. Are any (other) of your congregation members trained in health?

The 3rd element, access to intervention-related information, was assessed during the implementation phase and will be expanded on in that section.

(2) Implementation of the community health advocate (CHA) training programme

The implementation process is presented in two steps: (i) training and assessment of the CHAs and (ii) assessment of implementation determinants and outcomes (Fig. 1). Across the three sites, the process took place during the months of April to September 2018.

(i) Training and assessment of the CHAs: Once readiness of the faith-based organisation (FBO) was ascertained, all FBO members who volunteered were invited to the CHA training course. Volunteers indicated if they had any previous training in healthcare. The CHA course, which was taught by the study principal investigator with assistance from the medical doctor at the FBO, a dietician and an ethicist was carried out on the FBO compound and included didactic lectures and practical exercises in weight, blood pressure (BP) and blood glucose measurements, dietary history taking, electronic data entry, cooking and ethics (Table 2). This was followed by a simulation with volunteer ‘patients’ and a practical assessment by external examiners. Each skill was judged separately and CHAs had to show competence in order to be certified. The schedule required 2 h weekly for 10 weeks.
(ii) Assessment of implementation determinants and outcomes:Implementation effectiveness of the CHA training programme was determined from the success rates at the summative assessment. The odds of being successful at a particular station for persons with previous health care experience versus those without health care experience were also calculated. The statistical significance of the differences was explored using Fisher’s exact test.

**Table. 2:** The CHA training programme

Session	Core topics	Format	Mode of delivery	Resources
1	What I need to know about T2DMThe T2DM prevention, remission and referral protocolHow to measure waist & hip circumferences	Lecture, discussionpractical	Face to face, online resources	ComputerMeasuring tape
2	The physiology of T2DM remissionHow to measure waist & hip circumferences (repeat)How to perform blood pressure measurements	Lecture, discussionpractical	Face to face, online resources	Measuring tapeAutomated BP monitor
3	How to take a take a history including weekly food intake and medication adherenceHow to perform blood pressure measurements (repeat)How to perform a weight measurement	Practical	Face to face, online resources	Automated BP monitor, scale
4	How to perform a blood sugar using the point-of-care machineHow to use the RedCap database	Practical	Face to face, Online resources	Glucometer, computer
5.	How to perform a blood sugar using the point-of-care machine (repeat)How to perform a weight measurement (repeat)	Practical	Face to face, Online resources	Glucometer, scale
6.	Review of all practical demonstrations	Practical	Face to face, Online resources	All the above
7.	Simulation	Practical	Face to face	All the aboveVolunteer ‘patients’
8	Assessment	OSCE format	Face to face	All the aboveVolunteer ‘patients’
9.	Cookery	Practical	Face to faceOnline resources	Equipped kitchen, food
10.	Ethics	Case discussions	Face to face	

Four to 6 weeks after the completion of the CHA training, a survey was created in RedCap and distributed online via email to the CHAs. The survey consisted of 11 questions, formatted on a 5-point Likert scale, from strongly disagree to strongly agree. Seven questions assessed access to intervention-related information—the 3rd element in the assessment of readiness—by asking their perceptions of the content and delivery of the course material. Another four questions assessed their views of the attitude that potential clients might have towards the programme; this was mapped to the ‘Knowledge and beliefs about the intervention’ domain of the CFIR [29]. These scales demonstrated good measures of reliability with Cronbach’s alpha of 0.80 for the 7-point scale assessing intervention characteristics and 0.89 for the 4-point scale assessing attitude to the programme.

(3) Intervention: low-calorie diet (LCD) for eligible participants

Once the community health advocates (CHAs) were trained, a health event was held on the faith-based organisation (FBO) compound where interested persons could be screened for intervention eligibility. The invitation was initially extended to congregants and then a snowball effect was added. The intervention was a longitudinal study design with measurements recorded pre, during and post the low-calorie diet (LCD). The inclusion criteria were age 20–69 years, body mass index (BMI) ≥27 kg/m^2^ and a diagnosis of T2DM for <6 years or a diagnosis of pre-diabetes. The exclusion criteria were similar to those of the BDRS1 protocol, including those on insulin, pregnant or considering pregnancy, known serious illness or an HbA1c ≥12% [14]. Eligible persons went through the informed consent process. Potential participants who were eligible based on age and BMI but were unsure of their glucose status were also consented and then screened for glucose status using the fasting blood glucose (FBG), 2-h post prandial glucose and haemoglobin A1C (HbA1C). The American Diabetes Association (ADA) guidelines were used to determine eligibility that is those with 2 of the following results—FBG >7mmol/l, 2-h post prandial glucose > 11.1mmol/l and/or HbA1C >6.5%—would be diagnosed as T2DM [31]. Persons with 2 abnormal results including a FBG 5.6–6.9 mmol/L, 2-h post prandial glucose of 7.8–11 mmol/L and/or HbA1C of 5.7–6.4% would be diagnosed as pre-diabetes.

The daily dietary allowance was restricted to 840cal consisting of 4 bottles of Glucerna® (180cal per bottle (8oz))—which were provided free of cost to participants, 4 portions of non-starchy vegetables (30cal each) and at least 3L of non-calorie beverages. The intervention period was 12 weeks, with the FBO sites beginning in April, May and August 2019 consecutively. On the first day of the intervention all participants discontinued all anti-diabetic medication. Baseline blood pressure (BP), FBG and weights were done by their attending CHAs at the FBO and repeated weekly for the 12-week duration. HbA1C was done at week 12. A dietary history was also taken on a weekly basis to monitor compliance. Induction of remission was defined by the week 12 glucose measurements; for persons with T2DM, this was represented as an HbA1C <6.5% or a FBG of <7mmol/l while off of their anti-diabetes medication for the 12-week study duration [13]. For persons with pre-diabetes participants, remission was defined as normalisation of blood glucose, i.e. an HbA1C of <5.7% or a FBG of <5.6%.

The continuous variables of weight, HbA1C, FBG, systolic BP (SBP) and diastolic BP (DBP) and the change in these measurement at post intervention was analysed in Stata and presented as the mean (95% confidence interval). Individual level of analysis of these variables was performed and adjusted for clustering by church group. Differences by sex and by glucose status were also explored.

### Ethical considerations

The study was approved by the joint Ministry of Health/The University of the West Indies Institutional Review Board.

## Results

### Pre-implementation

Of the 3 faith-based organisations (FBOs) selected, 2 were located on the outskirts of Bridgetown—the capital of Barbados, while the third was located in a rural community. The average weekly attendance at the major weekly service was 240; women accounted for approximately 70% of the membership across the 3 sites.

#### Readiness

1. An engaged leadership

All 3 FBOs have taught on health-related issues at some time in the past; 2 sites had sessions in the last year—both had talks on cancer. In addition, one site organised a series of non-communicable disease (NCD)-related talks on diabetes, obesity, exercise and nutrition. The same site has an ongoing programme for prevention of NCDs in the form of a bi-weekly fitness group. All 3 sites indicated intentions of improving or implementing new health programmes but no strategic policies had been documented or enacted. Leaders at all sites were receptive to the idea of a diabetes prevention programme and reported perceptions that there was a high burden of NCDs in their community. Leaders also expressed feeling a responsibility to the congregation and communities to not only offer spiritual guidance but support towards physical health as well. They described this intervention as ‘timely’ and ‘necessary’.

2) Adequate resources

The leaders reported that the physical plants were adequate with private space for interviews and secure storage facilities. All but one of the facilities had a designated area for cooking activities. A site visit performed by the lead investigator confirmed these reports.

During the interviews, the organisational leaders across the 3 FBOs identified several health professionals amongst the congregation: 20 nurses, 7 physicians, 2 physiotherapists, 1 paramedic and 6 community health aides with care of the elderly experience; however, the sites had neither structured health programmes nor designated health leaders.

In response to the request for volunteers, 40 persons volunteered for training: 20 with no previous health experience, 9 nurses, 4 doctors, 3 first aiders, 1 pharmacist, 1 social worker, 1 paramedic and 1 community health aide.

### Implementation

#### Community health advocates

**Fig. 2:**
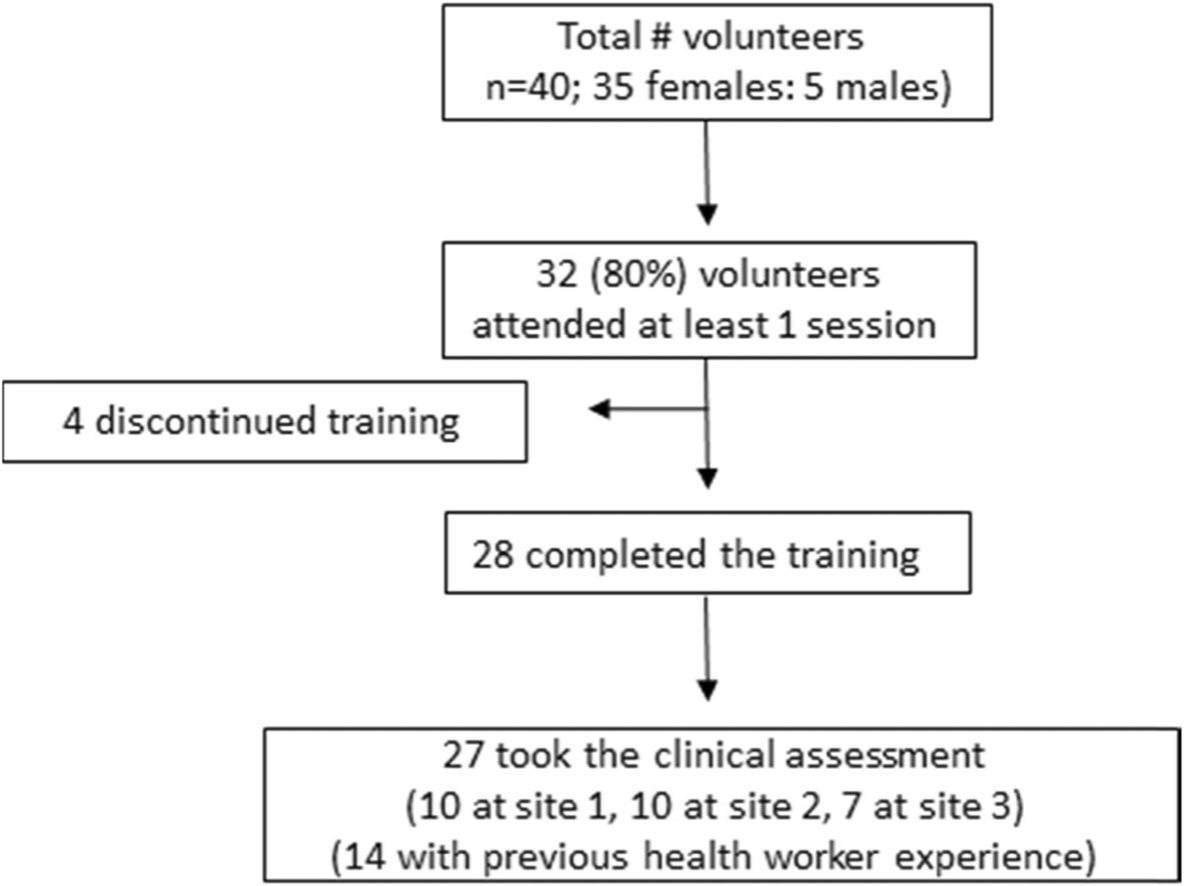
Flow diagram of community health advocates through the training process

Forty persons volunteered for the CHA training programme (35 females: 5 males); of these, 32 (80%) attended at least 1 session. Four of the 32 discontinued the programme during the first 2 weeks; the reasons were work commitments, family commitments, change in church membership and the programme was not what was expected. The remaining 28 (70%) completed the training and 27 (68%) of these took the clinical assessment; 10 at 2 sites and 7 at the third site. Of those 27, 14 had previous health worker experience including paramedics, nurses and clinicians (Fig. 2).

#### Implementation effectiveness

Amongst those who took the competence examination, the success rate for each station was BP (100%), weight (93%) [2 persons were not assessed and hence failed by default; all who were assessed passed], hip circumference (89%), waist circumference (78%) and glucose (74%). All CHA passed at least 1 station. The odds of success were better in persons with previous clinical training only at the glucometer station (OR=8.3, 1–224; *p*=0.05) (Table 3).

**Table. 3:** Percentage success rate at each clinical measurement station for community health advocates with previous health care experience vs those with no experience

	Experience (%)	No experience (%)	OR (95% CI)
Waist	85	71	2.1 (0.3, 19.8)
Hip	92	93	0.9 (0.02, 38.9)
Weight	100	85*	5.3 (0.4, 198.0)
Glucose	92	57	8.3 (1.0, 224)
BP	100	100	1

*2 (15%) persons did not take the test and hence failed by default

#### Easy access to intervention characteristics

Sixteen CHAs responded to the access to intervention-related information survey: 9 nurses, 2 of whom were retired and 2 in training; 3 persons with no health training, 2 with care of the elderly exposure, 1 paramedic and 1 first aider. Responses were condensed from the 5-point Likert scale (strongly agree, agree, neutral, disagree, strongly disagree) to a 3-point scale (agree, neutral, disagree) for easy reporting (Table 4). The majority of answers indicated that the content and delivery was adequate. One responder mentioned the need for additional dietary information including dietary allowances and menus. Another person thought that the time allotted to the course was inadequate especially for persons who had no previous health exposure; interestingly, all persons who had no previous exposure agreed that the time allotted to the course was adequate. One person disagreed that their knowledge and skills base was expanded while on this course; in each case, the person had previous health training.

**Table. 4:** Responses of CHAs on the content, delivery and acceptability of the intervention

Question	Agree *n* (%)	Neutral *n* (%)	Disagree *n* (%)
1. The content of the course was of an acceptable standard	15 (94)	0	1 (6)
2. The information was delivered in such a way that it was difficult to understand	1 (6)	0	15 (94)
3. The time allotted to the course was adequate	14 (88)	1 (6)	1 (6)
4. My knowledge base was expanded while on this course	14 (88)	1 (6)	1 (6)
5. My skills base was expanded while on this course	15 (94)	0	1 (6)
6. The course is good enough to be offered for a fee	13 (81)	3 (19)	0 (0)
7. I am likely to recommend this course to a friend or colleague should it become publicly available	16 (100)	0	0 (0)
8. Seeing participants according to the intervention schedule will disrupt the church schedule	0 (0)	3 (19)	13 (81)
9. Seeing participants according to the intervention schedule fits into my schedule	13 (81)	3 (19)	0 (0)
10. Utilising the church as the intervention site is convenient for participants	15 (94)	1 (6)	0 (0)
11. Utilising the church as the intervention site hinder participants from freely sharing information	0 (0)	2 (12)	14 (88)

#### Individuals’ attitudes to the intervention

CHAs unanimously agreed that the faith-based organisation (FBO) was a convenient and acceptable location for clients and that the schedule was not disruptive to the FBO or to themselves. This was confirmed by that attendance register which showed that all clinical measurement stations were covered by CHAs over the intervention period, barring one visit at one site during a church convention when the facility was closed and CHA and participants were away from the site.

### Intervention

**Fig. 3:**
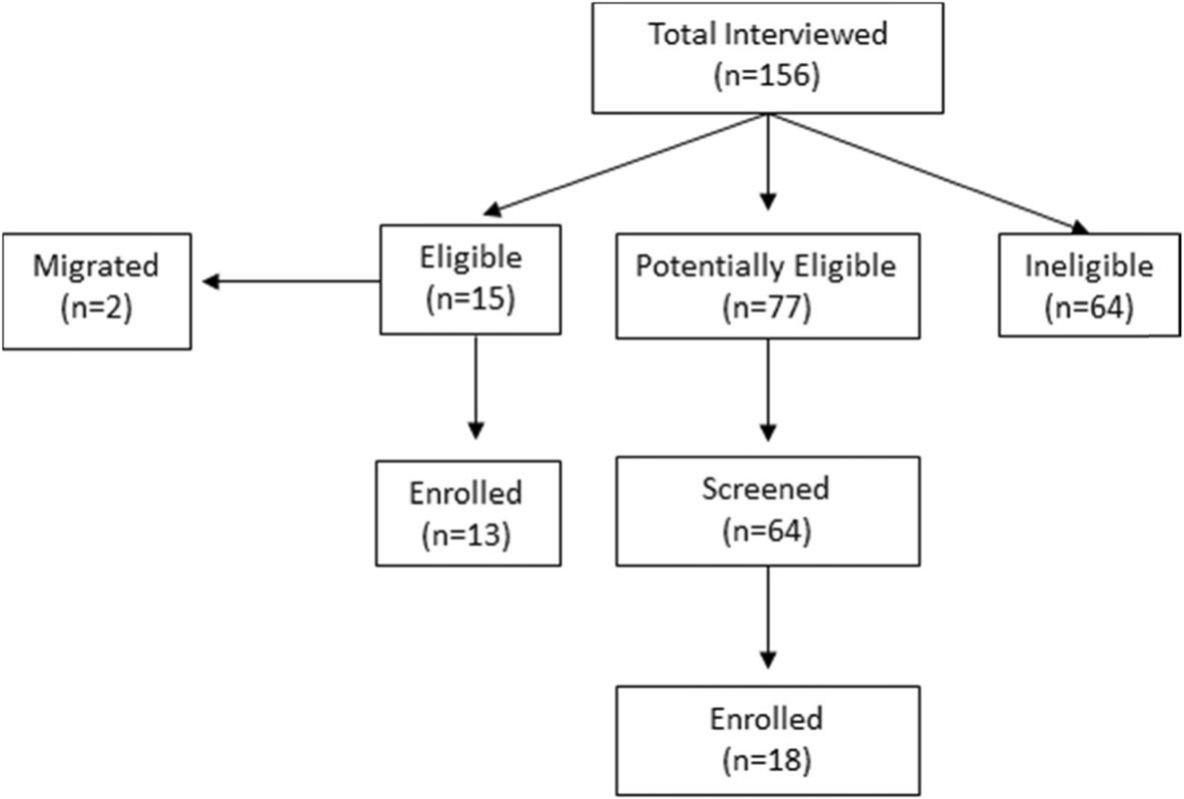
Flow diagram of participant recruitment

One hundred and fifty-six persons were interviewed over a 6-month period; 15 were immediately eligible; of these, 13 participated; the other 2 migrated. An additional 77 persons were potentially eligible based on age and BMI; 64 attended their appointments for screening for glucose status, and of these, 18 were enrolled (Fig. 3).

In all, 31 participants were enrolled across the 3 faith-based organisations (FBOs), the minimum enrolment per site was 6 participants, and one site enrolled 2 successive cohorts of 7 and 10 participants (Fig. 1). The age ranged from 36 to 66 years, all self-identified as being Black. Further recruitment was interrupted by the lockdown due to COVID19 pandemic. There were 28 (90%) females and 3 (10%) males, 11 (35%) with T2DM (10f: 1m), of whom 3 were diagnosed during screening for this study, all female. The 8 persons who were previously diagnosed were all on glucose-lowering medication; 7 on metformin; 3 of these in combination with a sulphonylurea and 2 with a dipeptidyl peptidase-4 (DPP-4) inhibitor; 1 person was on a sodium-glucose cotransporter-2 (SGLT-2) inhibitor alone. Twenty participants (65%) were enrolled with pre-diabetes, 13 were diagnosed during screening for this study (12f: 1m); 3 of the 7 who were previously diagnosed were on glucose-lowering medication; all 3 on metformin, 1 in combination with a DPP-4 inhibitor. Eleven participants had a history of hypertension (HTN); all were on anti-HTN medication. Nine participants had a history of high cholesterol, 4 were on HMG-CoA reductase inhibitors (Table 5).

**Table. 5:** Participant demographics

	n=31
Age on enrolment (years)	
Minimum–maximum	36–66
Sex	
Female	28 (90%)
Male	3 (10%)
Ethnicity	
Black	31 (100%)
Diabetes status	
Pre-diabetic, unaware	13 (42%)
Pre-diabetic, aware	7 (23%)
Type 2 diabetic, unaware	3 (10%)
Type 2 diabetic, aware	8 (26%)
Medication for diabetes (*n*=8)	
Metformin only	2
Metformin + sulphonylurea	3
Metformin + DPP-4* inhibitor	2
Sodium-glucose cotransporter-2 inhibitor	1
Medication for pre-diabetes (*n*=7)	
Metformin only	2
Metformin + DPP-4* inhibitor	1
None	4
Hypertension	11 (35%)
Medication for Hypertension (*n* = 11)	11
Hypercholesterolaemia	9 (29%)
Medication for Hypercholesterolaemia (*n*=9)	
HMG-CoA reductase inhibitors	4
None	5
*DPP-4-dipeptidyl peptidase-4	

Average baseline measurements adjusted for clustering by church were as such mean (95%CI): weight (kg) 94.8 (68.5 to 121.1); 92.5 in females vs. 116.0 in males; fasting blood glucose (FBG) (mmol/l) was 6.4 (5.7 to 7.1); 6.6 in T2DM vs. 6.3 in pre-diabetes and HbA1C (%) was 6.6 (6.1 to 7.1); and 6.1 and 7.4 in pre-diabetes and T2DM respectively (Table 6).

**Table. 6:** Change in weight, FBG and HbA1C post LCD interventions

	Pre-intervention Mean (95%CI)	Post-intervention Mean (95%CI)	Change (reduction) Mean (95%CI),
Weight (kg) [all]	94.8 (68.5 to 121.1)	88.8 (64.8 to 112.9)	6.0 (3.7 to 8.2)
Weight (kg) [male]	116.0 (113.4 to 118.7)	108.1 (102.6 to 113.7)	7.9 (−0.3 to 16.1)
Weight (kg) [female]	92.5 (66.8 to 118.2)	86.8 (63.7 to 109.9)	5.7 (3.1 to 8.4)
FBG (mmol/L) [all]	6.4 (5.7 to 7.1)	6.0 (5.3 to 6.7)	0.4 (−0.2 to 0.9)
FBG (mmol/L) male	6.4 (6.2 to 6.6)	6.6 (6.0 to 7.1)	−0.2 (−1.0 to 0.7)
FBG (mmol/L) female	6.4 (5.7 to 7.2)	6.0 (5.1 to 6.8)	0.4 (−0.0 to 0.9)
FBG (mmol/L) T2DM	6.6 (5.5 to 7.6)	6.4 (4.9 to 7.9)	0.2 (−0.5 to 0.8)
FBG (mmol/L) Pre-diab	6.3 (5.6 to 7.0)	5.8 (5.2 to 6.4)	0.5 (−0.1 to 1.2)
HbA1C (%) [all]	6.6 (6.1 to 7.1)	6.1 (5.7 to 6.5)	0.5 (0.1 to 0.8)
HbA1C (%) male	8.1 (3.0 to 13.1)	6.6 (6.1 to 7.2)	1.4 (−4.2 to 7.0)
HbA1C (%) female	6.4 (6.1 to 6.8)	6.0 (5.7 to 6.4)	0.4 (−0.4 to 1.1)
HbA1C (%) T2DM	7.4 (5.9 to 8.8)	6.3 (6.1 to 6.5)	1.2 (−0.7 to 3.0)
HbA1C (%) Pre-diab	6.1 (6.0 to 6.3)	6.0 (5.5 to 6.5)	0.1 (−0.1 to 0.4)

Mean (95%CI) change post-intervention was as such: weight loss 6.0 kg (3.7 to 8.2); 7.9 kg (− 0.3 to16.1) in males vs 5.7 kg (3.1 to 8.4) in females. FBG (mmol/l) reduction was 0.4 (−0.2 to 0.9); 0.2 (−0.5 to 0.8) in persons with T2DM and 0.5mmol/l (−0.1 to 1.2) in persons with prediabetes. HbA1C decreased by 0.5% (0.1 to 0.8), with a greater reduction in persons with T2DM 1.2 (−0.7 to 3.0) compared to those with prediabetes 0.1 (−0.1 to 0.4). Systolic blood pressure (SBP) and diastolic BP decreased by 10mmHg, *p*=0.003, and 8mmHg, *p*=0.005, respectively.

Of the 11 persons with T2DM who completed the low-calorie diet (LCD), 10 persons were available for FBG and HbA1C testing at week 12. Based on an HbA1C threshold of <6.5%, 6 (60%) were in remission. Based on the FBG of <7mmol/l, 9 (90%) were in remission.

Of the 20 persons with pre-diabetes, 17 completed the study and were available for HbA1C testing at week 12; only 15 were fasted and hence had FBG done. Based on an HbA1C threshold of <5.7%, 3 (18%) reverted to normal glucose status. Based on FBG, 6 (40%) reverted to normal glucose status (<5.6 mmol/L). One person achieved normal glucose status by both FBG and HbA1C.

Five of the 11 participants that were anti-hypertensive medications either decreased or discontinued the medication.

Review of the participant register showed high attendance throughout the study. Three participants prematurely discontinued the intervention; 1 at week 3 and 1 at week 10, both due to travel and another 1 at week 6 who was no longer interested in continuing; all 3 had pre-diabetes. The lowest attendance of 82% occurred during the convention week; apart form that, the lowest attendance was 89% at weeks 8 and 11. There were 21 absences in all (out of 429 visits); 1 absence occurred in 5 people, 2 in 2 people, 3 in 1 person, 4 in 1 person and 5 in 1 person.

## Discussion

### Pre-implementation

When assessing readiness of the faith-based organisations (FBO), we found high levels of leadership engagement and adequate resources. The act of involving the FBO leaders afforded them the opportunity to progress from acknowledging a need for an intervention to a place where they facilitated the intervention. If mapped to the transtheoretical model of change (TTM), action can be promoted via educational activities, particularly on the burden of disease within the population and the impact the intervention can make to the improvement in outcome [32]. The next step, long-term maintenance, which is defined by the continuation of the community health advocate (CHA)-led intervention beyond 6 months, can be facilitated by the provision of an environment that is conducive to the continuation of the intervention. In this study, maintenance has been facilitated as the glucometers, blood pressure kits, dietary booklets and other material that were provided during training will be available to the CHA for use after the study is completed. The TTM has been used to expound on the changes in individual behaviours across a wide range of practices including lifestyle modification for the management of non-communicable disease (NCDs) [33–35]. Within the recent scientific literature, the TTM has also been proposed as a framework for organisational change and community coalitions [36, 37]. More emphasis should be placed on leadership engagement within the community not simply as a facilitator of the intervention but as an agent of change.

### Implementation

The results suggest that the combination of trained CHAs within the context of a FBO is a useful implementation strategy in conducting a diabetes remission intervention. Theoretical barriers to implementation, such as human resources and infrastructural limitations, were not found to be present in the pre-implementation phase, where high levels of leadership engagement contributed to the state of readiness [38]. There was a high CHA retention rate, even in the face of a formal summative assessment prior to certification. The intervention-delivery training method was found to be acceptable and sufficiently thorough for 100% of trainees to achieve competence in ≥1 of the 5 clinical skills necessary to monitor the anthropometric and biochemical measures necessary. Extra attention, however, should be focused on glucometer competency in those with no previous healthcare experience. Despite reports of the effectiveness of CHA interventions in NCD management, there are concerns regarding unregulated CHA action and adverse patient outcome resulting from uncoordinated care [19, 39–41]. We were able to circumvent these critical issues by having a licensed medical practitioner as a member of the community to provide oversight to major clinical decisions.

The Chronic Care Model provides the evidence base for the growing recognition that NCD management requires integration of clinic-based and community-supported care. Whereas FBOs are being used increasingly as community hubs for health interventions, particularly in African descent populations, the results of the work are transferable on the theoretical level, where the FBOs represent “a safe space” to conduct the intervention and the CHAs are broadly categorised as “volunteers” who have the willingness and capacity to be trained [42, 43].

This transfer might necessitate some adaptation to the protocol. In framing the adaptation, one must decide what are the core elements that are necessary to maintain the fidelity of the intervention versus the peripheral components that can be adapted to increase the feasibility of the alternative design [44]. For e.g., to transfer the intervention from the FBO to a work site, it is decided that the 740-kcal diet for the 12-week duration are the core elements. However, congregants can be replaced by the safety officers as CHAs, clinical measurements can be assessed at the worksite instead of the FBO and participants can be offered a 740-kcal combination of solid and liquid meals instead of being restricted to the liquid formulation only.

### Intervention

Being female is a social determinant for obesity and development of T2DM in the Caribbean; gender-specific interventions are therefore warranted. In this study, 90% of participants were females; hence, FBO may be an ideal location to engage those most at risk.

Eighty percent of participants with pre-diabetes were newly diagnosed. Mathematical models predict that pre-diabetes to T2DM conversion occurs at approximately 2.5%/year, with persons of African and Asian descent being at higher risk [3, 43]. Pre-diabetes management hinges on weight loss, and a substantial amount of work has investigated the effects of various diets [45, 46]. In this study, participants with pre-diabetes were on average, able to surpass the recommended 5% reduction in body weight needed for T2DM risk reduction. In the absence of a community-based intervention, diagnosis may have been delayed or remained undiagnosed until symptomatic. We therefore recommend community-based screening for pre-diabetes and intensive dietary intervention for T2DM risk reduction and pre-diabetes remission.

Of the 10 persons with T2DM in this study, 3 reverted to normo-glycaemia, whereas the other seven achieved a reduction of glucose to pre-diabetes levels. Follow-up measurements would be useful to investigate the maintenance of this effect. Remission rates as defined by HbA1C were lower at 60% than by FBG (90%); this is in keeping with previous studies in both the Barbadian and the African American population which showed that for any given level of FBG, HbA1Cs levels tend be higher [47, 48].

Maintenance of weight loss is necessary to accrue maximum cardiovascular benefits. At the end of the 12-week low-calorie phase, participants transition to balanced, healthy meals under the guidance of a dietician. The curriculum includes learning to read food labels, trips to the supermarket and cooking classes. Although useful, continuing community-based lifestyle management including intensive dietary advice is necessary. This can be approached via two mechanisms: (1) training the CHAs in nutrition and dietetics which could potentially increase capacity and accessibility to dietary advice and (2) formalising relationships with surrounding health centres; this would allow for referral to trained dietician and any additional services, e.g. podiatry and ophthalmology; however, this avenue is limited by the availability of appointments.

## Conclusion

In conclusion, this feasibility study supports the proposal that weight loss due to a LCD can induce diabetes and pre-diabetes remission in a community context, as the FBO-based, CHA-supported implementation strategy was acceptable to all parties involved. A notable limitation of this study is that it was heavily subscribed by women; although gender-specific interventions are warranted, future studies could involve adaptation of this methodology to other community sites with the prospects of attracting a more balanced cohort. Although the small sample size may limit generalisability at the population level, the emerging themes are transferable on a theoretical level to other settings and may inform the scale up of community-based participatory research. Additional studies can consider how this protocol can be adapted to an online version to mitigate against interruptions due to social distancing and lockdown protocols.

## Data Availability

The datasets used and/or analysed during the current study are available from the corresponding author on reasonable request.

## References

[1] International Diabetes Federation (IDF). Diabetes Atlas. 9th Edition. Belgium: International Diabetes Federation; 2019.

[2] Crandall JP, Knowler WC, Kahn SE, Marrero D, Florez JC, Bray GA (2008). The prevention of type 2 diabetes. Nat Clin Pract Endocrinol Metab.

[3] Yokota N, Miyakoshi T, Sato Y, Nakasone Y, Yamashita K, Imai T, Hirabayashi K, Koike H, Yamauchi K, Aizawa T (2017). Predictive models for conversion of prediabetes to diabetes. J Diabetes Complications.

[4] Kim YA, Ku EJ, Khang AR, Hong ES, Kim KM, Moon JH, Choi SH, Park KS, Jang HC, Lim S (2014). Role of various indices derived from an oral glucose tolerance test in the prediction of conversion from prediabetes to type 2 diabetes. Diabetes Res Clin Pract.

[5] Leandro CG, da Fonseca E, de Lim CR, Tchamo ME, Ferreira ESWT (2019). Barriers and enablers that influence overweight/obesity/obesogenic behavior in adolescents from lower-middle income countries: a systematic review. Food Nutr Bull.

[6] Mancini MC, Halpern A (2008). Orlistat in the prevention of diabetes in the obese patient. Vasc Health Risk Manag.

[7] Buchanan TA, Xiang AH, Peters RK, Kjos SL, Marroquin A, Goico J, Ochoa C, Tan S, Berkowitz K, Hodis HN, Azen SP (2002). Preservation of pancreatic β-cell function and prevention of type 2 diabetes by pharmacological treatment of insulin resistance in high-risk hispanic women. Diabetes..

[8] World Health Organization. Obesity and Overweight. 2016. http://www.who.int/mediacentre/factsheets/fs311/en.

[9] Pan American Health Organization. NCDs at a Glance: NCD Mortality and Risk Factor Prevalence in the Americas. Washington, D.C.: PAHO; 2019.

[10] Guariguata L, Brown C, Sobers N, Hambleton I, Samuels TA, Unwin N (2018). An updated systematic review and meta-analysis on the social determinants of diabetes and related risk factors in the Caribbean. Rev Panam Salud Publica.

[11] Batterham RL, Cummings DE (2016). Mechanisms of diabetes improvement following bariatric/metabolic surgery. Diabetes Care.

[12] Sjöström L, Lindroos AK, Peltonen M, Torgerson J, Bouchard C, Carlsson B, Dahlgren S, Larsson B, Narbro K, Sjöström CD, Sullivan M, Wedel H (2004). Lifestyle, diabetes, and cardiovascular risk factors 10 years after bariatric surgery. N Engl J Med.

[13] Lim EL, Hollingsworth KG, Aribisala BS, Chen MJ, Mathers JC, Taylor R (2011). Reversal of type 2 diabetes: normalisation of beta cell function in association with decreased pancreas and liver triacylglycerol. Diabetologia..

[14] Bynoe K, Unwin N, Taylor C, Murphy MM, Bartholomew L, Greenidge A, Abed M, Jeyaseelan S, Cobelli C, Dalla Man C, Taylor R. Inducing remission of Type 2 diabetes in the Caribbean: findings from a mixed methods feasibility study of a low-calorie liquid diet-based intervention in Barbados. Diabet Med. 2020;37(11):1816–24. 10.1111/dme.14096. Epub 2019 Aug 12. 10.1111/dme.14096 31365159

[15] Taylor R, Leslie WS, Barnes AC, Brosnahan N, Thom G, McCombie L, Sattar N, Welsh P, Peters C, Zhyzhneuskaya S, Hollingsworth KG, al-Mrabeh A, Rodrigues AM, Rehackova L, Adamson AJ, Sniehotta FF, Mathers JC, Ross HM, McIlvenna Y, Kean S, Ford I, McConnachie A, Lean MEJ (2018). Clinical and metabolic features of the randomised controlled Diabetes Remission Clinical Trial (DiRECT) cohort. Diabetologia..

[16] Balagopal P, Kamalamma N, Patel TG, Misra R (2012). A community-based participatory diabetes prevention and management intervention in rural India using community health workers. Diabetes Educ.

[17] McCloskey J (2009). Promotores as partners in a community-based diabetes intervention program targeting Hispanics. Fam Community Health.

[18] Joshi R, Alim M, Kengne AP, Jan S, Maulik PK, Peiris D, Patel AA (2014). Task shifting for non-communicable disease management in low and middle income countries--a systematic review. PLoS One.

[19] Jeet G, Thakur JS, Prinja S, Singh M (2017). Community health workers for non-communicable diseases prevention and control in developing countries: Evidence and implications. PLoS One.

[20] Schoenthaler AM, Lancaster KJ, Chaplin W, Butler M, Forsyth J, Ogedegbe G (2018). Cluster randomized clinical trial of FAITH (Faith-Based Approaches in the Treatment of Hypertension) in blacks. Circ Cardiovasc Qual Outcomes.

[21] Lancaster KJ, Carter-Edwards L, Grilo S, Shen C, Schoenthaler AM (2014). Obesity interventions in African American faith-based organizations: a systematic review. Obes Rev.

[22] World Health Organization. Noncommunicable diseases country profiles 2018. World Health Organization. 2018. https://apps.who.int/iris/handle/10665/274512.

[23] Unwin N, Rose AMC, George KS, Hambleton IR, Howitt C (2015). The Barbados health of the nation survey: core findings. Miller Publishing Company.

[24] Barbados Government Information System. Preventing & Controlling Type 2 Diabetes. 2016. https://gisbarbados.gov.bb/blog/preventing-controlling-type-2-diabetes/.

[25] Healthy Caribbean Coalition. Faith-based collaboration to counteract chronic diseases. DECLARATION-OFBridgetown-FBO-NCDS-FEB-26-2014.pdf (https://healthycaribbean.org).

[26] Samuels TA, Unwin N (2018). The 2007 Caribbean Community Port-of-Spain Declaration on noncommunicable diseases: an overview of a multidisciplinary evaluation. Revista panamericana de salud publica =. Pan American journal of public health.

[27] Landes SJ, McBain SA, Curran GM (2019). An introduction to effectiveness-implementation hybrid designs. Psychiatry Res.

[28] Villalobos Dintrans P, Bossert TJ, Sherry J, Kruk ME (2019). A synthesis of implementation science frameworks and application to global health gaps. Glob Health Res Policy.

[29] Damschroder LJ, Aron DC, Keith RE, Kirsh SR, Alexander JA, Lowery JC (2009). Fostering implementation of health services research findings into practice: a consolidated framework for advancing implementation science. Implementation science : IS.

[30] Department of Public Information. New group looking to target Non-Communicable Diseases through religious organisations -Minister Harmon pledges Government’s support – Department of Public Information (dpi.gov.gy). 2017.

[31] Classification and Diagnosis of Diabetes: Standards of Medical Care in Diabetes—2018. American Diabetes Association. Diabetes Care. 2018;41(Supplement 1):S13–27. 10.2337/dc18-S002. 10.2337/dc18-S002 29222373

[32] Abdel-All M, Putica B, Praveen D, Abimbola S, Joshi R (2017). Effectiveness of community health worker training programmes for cardiovascular disease management in low-income and middle-income countries: a systematic review. BMJ Open.

[33] Mastellos N, Gunn LH, Felix LM, Car J, Majeed A. Transtheoretical model stages of change for dietary and physical exercise modification in weight loss management for overweight and obese adults. Cochrane Database Syst Rev. 2014;(2):CD008066. 10.1002/14651858.CD008066.pub3. 10.1002/14651858.CD008066.pub3 PMC10088065 24500864

[34] Marcus BH, Sismkin LR (1994). The transtheoretical model: applications to exercise behavior. Med Sci Sports Exerc.

[35] Tseng HM, Liao SF, Wen YP, Chuang YJ (2017). Stages of change concept of the transtheoretical model for healthy eating links health literacy and diabetes knowledge to glycemic control in people with type 2 diabetes. Prim Care Diabetes.

[36] Prochaska JM, Prochaska JO, Levesque DA (2001). A transtheoretical approach to changing organizations. Adm Policy Ment Health.

[37] Finnegan HA, Langhinrichsen-Rohling J, Blejwas E, Hill A, Ponquinette D, Archer S, Kelley M, Allison M (2018). Developing a productive workgroup within a community coalition: transtheoretical model processes, stages of change, and lessons learned. Progress in community health partnerships : research, education, and action.

[38] Quimby KR, Jordan T, George C, Sobers N, Hambleton I. Assessing the readiness of faith-based organisations as sites for the Barbados Diabetes Remission Study 2 – a community-based diabetes remission intervention. West Indian Med J. 2019;68(Suppl. 1):70.

[39] Hughes MM, Yang E, Ramanathan D, Benjamins MR (2016). Community-based diabetes community health worker intervention in an underserved Chicago population. J Community Health.

[40] Brown LD, Vasquez D, Salinas JJ, Tang X, Balcazar H (2018). Evaluation of healthy fit: a community health worker model to address hispanic health disparities. Prev Chronic Dis.

[41] van de Ruit C (2019). Unintended consequences of community health worker programs in South Africa. Qual Health Res.

[42] Dodani S, Sullivan D, Pankey S, Champagne C (2011). HEALS: a faith-based hypertension control and prevention program for African American churches: training of church leaders as program interventionists. Int J Hypertens.

[43] Fazli GS, Moineddin R, Bierman AS, Booth GL (2020). Ethnic variation in the conversion of prediabetes to diabetes among immigrant populations relative to Canadian-born residents: a population-based cohort study. BMJ Open Diabetes Res Care.

[44] Miller CJ, Wiltsey-Stirman S, Baumann AA (2020). Iterative Decision-making for Evaluation of Adaptations (IDEA): a decision tree for balancing adaptation, fidelity, and intervention impact. J Community Psychol.

[45] Guess ND (2018). Dietary interventions for the prevention of type 2 diabetes in high-risk groups: current state of evidence and future research needs. Nutrients..

[46] Amer OE, Sabico S, Alfawaz HA, Aljohani N, Hussain SD, Alnaami AM (2020). Reversal of prediabetes in Saudi adults: results from an 18 month lifestyle intervention. Nutrients..

[47] Unwin N, Howitt C, Rose AM, Samuels TA, Hennis AJ, Hambleton IR (2017). Prevalence and phenotype of diabetes and prediabetes using fasting glucose vs HbA1c in a Caribbean population. J Glob Health.

[48] Herman WH, Ma Y, Uwaifo G, Haffner S, Kahn SE, Horton ES, Lachin JM, Montez MG, Brenneman T, Barrett-Connor E, for the Diabetes Prevention Program Research Group (2007). Differences in A1C by race and ethnicity among patients with impaired glucose tolerance in the Diabetes Prevention Program. Diabetes Care.

